# Statistical Design and Optimization of Cr (VI) Adsorption onto Native and HNO_3_/NaOH Activated Cedar Sawdust Using AAS and a Response Surface Methodology (RSM)

**DOI:** 10.3390/molecules28217271

**Published:** 2023-10-25

**Authors:** Maryam El Hajam, Noureddine Idrissi Kandri, Sadin Özdemir, Gabriel Plavan, Naoufel Ben Hamadi, Fehmi Boufahja, Abdelaziz Zerouale

**Affiliations:** 1 School of Forest Resources and Advanced Structures and Composites Center, University of Maine, Orono, ME 04469, USA; maryam.el1@maine.edu; 2 Processes, Materials and Environment Laboratory (PMEL), Faculty of Sciences and Techniques, Sidi Mohammed Ben Abdellah University, Road Imouzzer, Fez BP 2202, Morocco; azerouale@yahoo.fr; 3 Signals Systems and Components Laboratory (SSCL), Faculty of Sciences and Techniques, Sidi Mohammed Ben Abdellah University, Road Imouzzer, Fez BP 2202, Morocco; noureddine.idrissikandri@usmba.ac.ma; 4 Food Processing Programme, Technical Science Vocational School, Mersin University, Mersin 33343, Turkey; sadinozdemir@mersin.edu.tr; 5 Department of Biology, Faculty of Biology, Alexandru Ioan Cuza University, Bvd. Carol I. No. 20A, 700505 Iasi, Romania; gabriel.plavan@uaic.ro; 6 Chemistry Department, College of Science, Imam Mohammad Ibn Saud Islamic University (IMSIU), Riyadh 11623, Saudi Arabia; nabenhamadi@imamu.edu.sa; 7 Biology Department, College of Science, Imam Mohammad Ibn Saud Islamic University (IMSIU), Riyadh 11623, Saudi Arabia

**Keywords:** Cr (VI) removal, cedar sawdust, chemical activation, batch adsorption, response surface methodology, optimization

## Abstract

The removal of heavy metals from wastewater has become the subject of considerable interest at present. Thus, the use of novel adsorbents that are highly efficient is of critical importance for the removal of Cr (VI) ions from aqueous media. The adsorption of Cr (VI) ions from aqueous solutions by a new adsorbent, cedar wood sawdust, and the optimization of its adsorption parameters, were investigated in this study. Cedar wood sawdust was used in its native and HNO_3_/NaOH chemically modified forms as new low-cost sorbents to remove Cr (VI) ions from aqueous solutions in a batch system. The adsorption conditions were analyzed via response surface methodology. The RSM results showed that the optimal adsorption conditions yielding the best response were an adsorbent mass of 2 g for native Cedar and 1.125 g for its activated form, a metal concentration of 150 mg/L for native Cedar and 250 mg/L for activated, a temperature of 50 °C, a pH of 1, and a contact time of 67.5 min. At optimum adsorption conditions, the maximum adsorption capacities and the adsorption yields were 23.64 mg/g and 84% for native Cedar and 48.31 mg/g and 99% for activated Cedar, respectively.

## 1. Introduction

Due to the rapid development of industrialization over the last century, the discharge of heavy metals in industrial effluents such as from mining, battery manufacturing, metallurgy, and paints, has resulted in serious concerns from scientists and engineers because of their supreme toxicity to human health and ecological systems [1,2,3,4]. Unlike most organic pollutants, heavy metals are generally refractory and non-readily detoxified biologically.

Chrome is one of the heavy metal ions extensively used in diverse industries for making different products. However, chrome contamination is considered a serious environmental pollutant; it is a metabolic and non-degradable poison, and enzyme inhibitor, and it damages DNA, resulting in chromosomal and nuclear aberrations [5]. Chrome pollutant toxicity is determined by its vacancy states; hexavalent chrome is highly noxious, oncogenic, and transportable, and exists in a dichromate state in acidic and a chromate state in alkaline conditions, whereas Cr (III) is less toxic. According to the Environmental Protection Agency (EPA), the chrome concentration in drinking water is <0.1 ppm [6].

Therefore, the removal of chrome from such industrial effluents is a challenging requirement for producing a safe and clean environment. Several techniques have been reported for the removal of chrome (VI) such as magnetic flocculation [7], the exchange of ions [8], chemical precipitation [9], solvent extraction [10], and adsorption [11]. Among these techniques, adsorption has generated considerable interest in recent years and is becoming the most popular method due to its low cost, simplicity, better performance, low sludge generation, no production of secondary compounds that might be toxic, and the availability of various kinds of adsorbents [12]. The use of adsorbents based on agricultural by-products as lignocellulosic materials, such as wood sawdust [13], beet pulp [14], palm kernels [15], and rice husks [16], for the removal of heavy metals from wastewater has attracted intense attention because they are easily available, abundant in nature, inexpensive and environmentally friendly. These natural biomasses form complexes with metal ions using the ligand or functional groups existing in their proteins, carbohydrates, and phenolic compounds, which include carboxyl, hydroxyl, sulfate, phosphate, and amino groups exhibiting specific affinity to metal ions and thus can bind them. It has been confirmed that the reuse of wastes from agricultural products for the treatment of wastewater is an attractive and promising option with a double benefit for the environment [17]:

1. It reduces the solid residues, of which disposal methods and costs constitute a major problem and;
2. It gives a new life to these wastes by converting them into useful and inexpensive decontaminants for water purification.

Atlas cedar (*Cedrus atlantica Manetti*) is among the most important softwood (coniferous) species of the Pinaceae of North Africa and is native to the Atlas Mountains of Algeria and Morocco [18,19]. It covers about 160,000 ha, mostly in the Rif and Atlas Mountains of Morocco. The height of the trees can reach 40 m with trunk diameters of 2 m. It is well-known for its noble timber, which is highly sought after for construction [20]. Actually, Atlas cedar is the main species in Moroccan forests used for timber production, and the sawdust produced during wood processing is often used and highly regarded in energy fields, which generate environmental pollution. For this reason, we are interested in the sustainable and environmental aspects of wood waste, through the revaluation of Atlas Cedar sawdust as an adsorbent in its native and modified states for the adsorption of chrome (VI).

Many factors can affect the adsorption rate of metallic ions on native and modified sawdust, such as the adsorbent dosage, initial metal ion concentration, temperature, pH, and contact time. Traditionally, the optimization has been carried out using a mono-variate process; based on this conventional method, only the parameter to be tested is varied, while the others are fixed at a certain value. However, this method has many inconveniences as it involves a variety of experiments that take a long time and require large quantities of chemicals, and as it is not able to define the interaction between the parameters examined; it cannot provide statistical data to explain the details of the impact of the parameters on the response [21]. Therefore, it is important to select an appropriate optimization procedure, which can evaluate the influence of critical parameters along with possible interactions, using the fewest experiments, as Bhunia and Ghangrekar have suggested [22].

Experimental design methodology is a more powerful technique, which has been widely used to overcome the shortcomings of the conventional approach, and explains the interactive effect between the parameters. Recently, the response surface methodology (RSM) has become one of the strongest optimization and modeling procedures [23,24,25,26]. The response surface methodology (RSM) hinges on a central composite design (CCD) and is based on a combination of mathematical algorithms and statistical techniques using linear or quadratic polynomial functions [27]. It is a useful method for evaluating the effects of various factors, influencing the responses by varying them simultaneously and considering only the significant ones, which leads to performing fewer experiments and therefore taking less time and reducing costs [28,29,30]. CCD usually involves three steps: (1) performing the designed experiments, (2) estimating the coefficients in a mathematical model, and (3) optimization.

Therefore, the objectives of the present work are as follows: (1) the assessment of the potential of native and HNO_3_/NaOH-modified Cedar sawdust to adsorb Cr (VI) ions from aqueous solutions by studying several parameters, (2) the characterization of the native and modified biosorbent using DRX, FTIR, and SEM in order to provide relationships between its structure and adsorption capacity of Cr (VI) ions, and (3) the application of a central composite design (CCD) under the response surface methodology (RSM) approach to optimize the various operating parameters (adsorbent dosage (m), chrome (VI) concentration (Co), temperature (T), pH, and contact time (t)), and to analyze equilibrium data using different isotherm and kinetic models. 

## 2. Results and Discussion

### 2.1. Characterization of Biosorbents

#### 2.1.1. Scanning Electron Microscopy (SEM)

**Figure 1:**
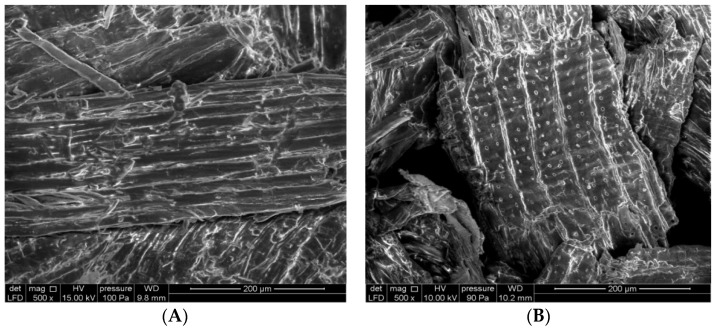
SEM photos of cedar sawdust; (**A**): native form (**B**): modified with HNO_3_/NaOH.

Morphological analysis via the scanning electron microscopy of native (Figure 1A) and chemically activated cedar sawdust (Figure 1B) shows significant changes in the topography of their surfaces. This could be explained by the beginning of cellulosic defibrillation because of the dissolution of extractives, hemicelluloses, and a large part of lignin, thus leading to a reduction in the lengths of the microfibrils and to an improvement in the porosity of these sawdusts (Figure 1).

#### 2.1.2. X-ray Diffraction (XRD)

**Figure 2:**
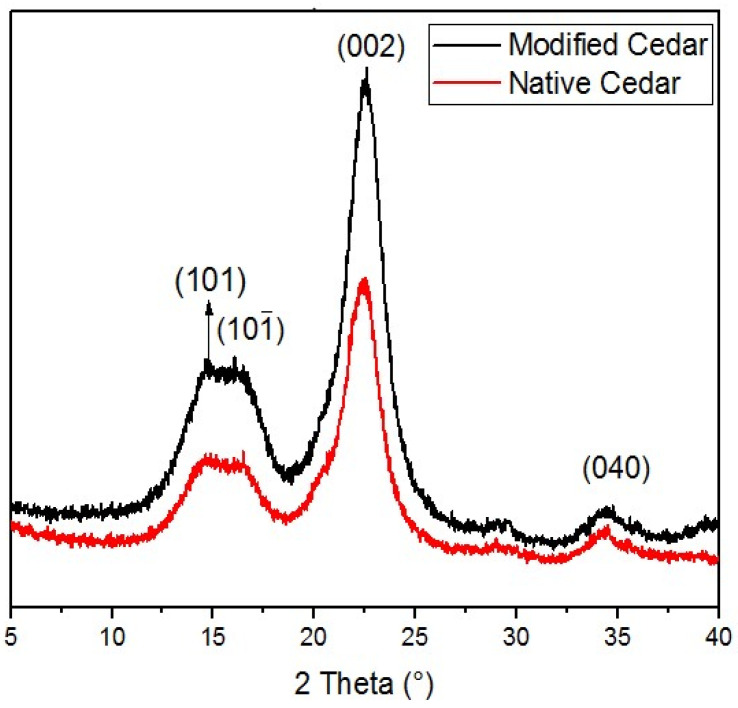
Diffractograms of native cedar sawdust and sawdust chemically modified with HNO_3_/NaOH.

As in the native state, the diffractograms of chemically activated cedar sawdust show four characteristic peaks of cellulose I, located around 2θ = 14°, 16°, 22°, and 34°, corresponding to the crystallographic planes (101), (002), and (040), respectively (Figure 2) [31]. After chemical activation, we notice that the intensity of these peaks increases, thus leading to an increase in the crystallinity index which rises from 41.24% to 73.53%.

#### 2.1.3. Infrared Absorption Spectroscopy (IRTF)

**Figure 3:**
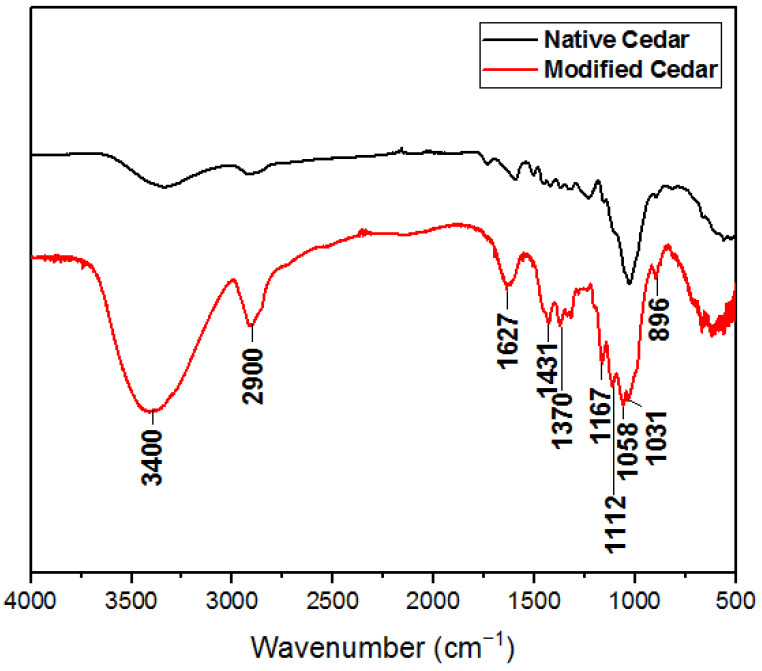
Infrared spectrum of native and HNO_3_/NaOH-modified cedar sawdust.

From the absorption spectra, it can be seen that the chemical treatment of sawdust with nitric acid followed by sodium hydroxide leads to the disappearance of the absorption band characteristic of the valence vibration of C=O of carboxylic acids and/or xylan esters present in hemicelluloses and lignin (around 1725 cm^−1^), as well as the disappearance of the absorption band attributed to the C=C deformation of the aromatic rings of lignin and the vibration of the CH bonds of aromatic polysaccharides (around 1500 cm^−1^). There is also a decrease in the intensity of the absorption bands linked to the deformation of the C-H bonds of lignin and hemicelluloses due to the solubilization of most lignin and hemicelluloses (between 1230 and 1370 cm^−1^) [32]. On the other hand, the characteristic bands of cellulose (1167 cm^−1^; 1112 cm^−1^, and 1058 cm^−1^) become sharper (Figure 3).

### 2.2. Adsorbent Performance Study toward Cr (VI) Adsorption Experiments

#### 2.2.1. Experimental Design and Data Analysis via RSM

RSM/CCD experiments were performed to designate the maximum %Cr (VI) adsorption and to optimize the effects of the parameters investigated on the removal of Cr (VI), including the adsorbent mass (X1), initial concentration of Cr (VI) (X2), temperature (X3), pH (X4), and contact time (X5). The corresponding responses (%Cr (VI) adsorption) for each experiment are shown in the experiment program created via RSM/CCD, using NemrodW software version 2007-03 (Table 1). The coefficients of the model for the responses were appraised using multiple regression analysis methods based on Equation (6). The empirical relationship between Cr (VI) adsorption onto native cedar and modified cedar, and the input parameters, are expressed using the following quadratic models, respectively:

%Ads of Cr (VI)/native Cedar = 58.300 + 7.167 X_1_ − 5.361 X_2_ + 8.778 X_3_ − 16.750 X_4_ + 1.639 X_5_ − 14.462 (X_4_ × X_4_) + 0.313 (X_1_ × X_2_) + 1.625 (X_1_ × X_5_) − 2.688 (X_2_ × X_5_) (1)

%Ads of Cr (VI)/modified Cedar = 80.776 + 5.000 X_1_ − 6.028 X_2_ + 9.361 X_3_ − 18.750 X_4_ + 2.806 X_5_ − 20.311 (X_3_ × X_3_) − 19.811 (X_4_ × X_4_) − 3.311 (X_5_ × X_5_) + 1.438 (X_1_ × X_2_) + 1.063 (X_1_ × X_5_) − 1.000 (X_2_ × X_5_) (2)

The value of the coefficient in Equations (1) and (2) indicates the intensity, and its sign indicates the positive or negative effect of the input parameter on the Cr (VI) adsorption. The positive influence of a factor means that when the factor level increases, the Cr (VI) removal is improved and vice versa [33]. According to Equations (1) and (2), the effective factors for Cr (VI) removal are as follows:

1. In the case of native cedar: pH, temperature, contact time, adsorbent mass, and initial concentration of Cr (VI), and the interaction of two variables—initial concentration and contact time—are the most important.
2. In the case of modified cedar: pH, temperature, contact time, initial concentration of Cr (VI), and adsorbent mass, and the interaction of two variables—adsorbent mass and initial concentration—are the most important.

The results of the quadratic model in terms of analysis of variance (ANOVA) for Equations (1) and (2) are summarized in Table 2.

As is known, the *p*-value gives an idea of the significance level of the variables, as the smaller the value of *p*, the more significant the corresponding coefficient term [33]. As shown in Table 3, the *p*-values less than 0.05 indicate that the model is significant; it is also observed that X_1_, X_2_, X_3_, X_4_, X_5_, X_4_X_4_, X_1_X_2_, X_1_X_5_, and X_2_X_5_ are significant model terms for both forms of cedar, in addition to X_3_X_3_ and X_5_X_5_ in the case of modified cedar only. 

#### 2.2.2. Statistical Analysis and Validation of the Model

The analysis of variance (ANOVA) is considered essential to test the statistical significance of a model through assessing the “goodness of fit”, and a lack of fit describes the variation of the data around the fitted model [34,35]. The statistical significance of the quadratic model evaluated via ANOVA is shown in Table 3. It is determined by the corresponding *p*-value, sum of squares (SS), degree of freedom (DF), and coefficient of determination (R^2^ and R^2^ adj). In general, a low probability ‘P’ value indicates the high significance of the regression model [30]. The sum of squares should also be checked when considering the significance of a particular variable [36,37]. When the value of SS increases, the significance of that variable also increases. The quality of the polynomial model was also expressed by the coefficient of determination (R^2^ and R^2^adj). R^2^ is a measure of the amount of variations around the mean explained by the model. The adjusted R^2^ is adjusted for the number of terms in the model, and it decreases as the number of terms in the model increases, if those additional terms do not add value to the model [38]. The values of R^2^ and R^2^Adj should be high for a good fit. 

It is clear from the ANOVA results (Table 3) that the model was highly significant and meaningful with a very low probability value (*p* < 0.01) and high SS for all responses, which can appropriately explain the relationship between each response and independent variable. The smaller the *p*-value (*p* < 0.05), the more significant the corresponding term, which indicates the rejection of the null hypothesis. Generally, *p* values lower than 0.01 indicate that the model is considered to be statistically significant at the 99% confidence level [39]. The quadratic model has a fairly high determination coefficient (R^2^ and R^2^ Adj), which indicates that the model fits the experimental data well for all samples. In the case of Cr (VI) removal using native cedar, the coefficient of determination R^2^ is 0.82, and the adjusted coefficient of determination R^2^ Adj. is 0.74. In the case of Cr (VI) removal using modified cedar, the coefficient of determination R^2^ is 0.88, and the adjusted coefficient of determination R^2^ Adj. is 0.78. 

**Figure 4:**
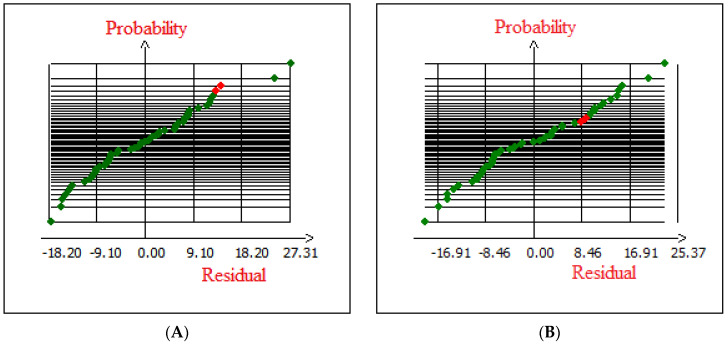
Normal probability distribution of residual (**A**) native cedar and (**B**) modified cedar.

**Figure 5:**
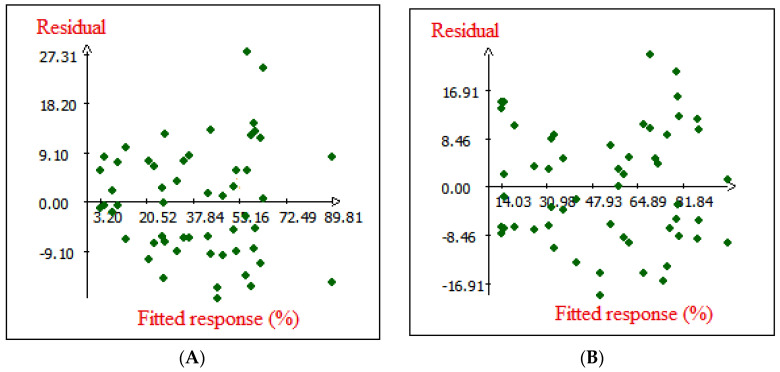
Residuals versus fitted response of (**A**) native cedar and (**B**) modified cedar.

Figure 4 shows the plot of observed Cr (VI) removals on native and modified cedar versus those obtained from the model, which will later reveal the comparison between these two. The results obtained indicate a high dependence and correlation between the observed experimental and predicted data of the responses (Cr (VI) removal onto native and modified cedar sawdust) via the empirical quadratic model. Because the curve is quite straight, the fit of the predicted data in the quadratic model for Cr (VI) removal onto the experimentally observed data for native and modified Cedar is quite good. Furthermore, the examination of the obtained fitted quadratic model is important. If this later fails to reveal an adequate correlation, the model optimization methods will probably generate erroneous or unwanted predictions. In this matter, the residuals have an essential role in evaluating the suitability of the model. As shown in Figure 5, the residuals did not distribute regularly along the zero line; however, the distribution of data was observed between approximately ±9.10 in the case of native cedar and ±16.91 in the case of modified cedar. These results show that the observed experimental data agrees quite well with the data predicted by the empirical quadratic model [40].

#### 2.2.3. Optimization of Studied Parameters via the CCD of RSM

As mentioned above, regarding the effect of factors on the adsorption of Cr (VI) (Table 2), pH, temperature, contact time, adsorbent mass, and the initial concentration of Cr (VI), and the interactions between adsorbent mass/initial concentration, adsorbent mass/contact time, and initial concentration/contact time are the key parameters that significantly affect the adsorption percentage of Cr (VI) ions from aqueous solutions. 

pH has a negative effect on the removal of Cr (VI), i.e., the adsorption rates of Cr (VI) decrease as the pH of the reaction medium increases. By rising from a pH of 1 to a pH of 6, the adsorption rates, respectively, pass from 77% to 22% for native cedar sawdust and from 98% to 32% for activated cedar sawdust. According to these results and based on the diagram of the distribution of the different chromic species as a function of pH, we can say that in a pH interval ranging from 1 to 3, the levels of Cr (VI) adsorption are at a maximum. Highly acidic pHs cause an increase in hydronium ions (H^+^), thus promoting the adsorption of Cr (VI), which is in its anionic form (HCrO_4_^−^) [41]. However, when the pH is increased, the HCrO_4_^−^ ions change to other forms, chromates (CrO_42_^−^) or dichromates (Cr_2_O_72_^−^), and the solution becomes charged with hydroxyl ions (OH^−^) which compete with the metal ions formed for the active sites of adsorption [42]. This allows us to observe that hexavalent chrome can only be adsorbed on native and activated cedar sawdust if it is in its monoanionic form HCrO_4_^−^. Similar results were reported by Ramos, R.L. et al. [43].

The initial concentration of Cr (VI) also has a negative effect on the removal rates of the Cr (VI); the adsorption percentages reach their maximum for Cr (VI) concentrations ranging from 10 to 50 mg/L for native cedar and from 10 to 100 mg/L for the activated sawdust. With a concentration of 250 mg/L, the adsorption rates drop from 69% to 38% for native cedar sawdust and from 92% to 75% for activated cedar. It is noted that activated sawdust has a greater adsorbent power exceeding that of native sawdust by approximately 22% when the initial concentration is 10 mg/L and by approximately 35% at 250 mg/L. The reduction in adsorption rates when 50 mg/L is exceeded in the case of native sawdust and 100 mg/L in the case of activated sawdust can be explained by the reduction in the number of functional adsorption sites. Similar results were reported by Ucun, H. et al. and by Park, D. et al. [44,45].

The contact time has a positive effect on the adsorption rates of Cr (VI) on both native and chemically activated sawdust, which go through three stages:

1. The first stage is characterized by very rapid adsorption during the first 30 min in the case of native sawdust with an average adsorption rate of 55%, and during the first 25 min for activated sawdust with an average adsorption rate of 70%.
2. In the second stage, the adsorption becomes increasingly slow for both sawdusts.
3. The third stage is characterized by the establishment of a level that illustrates the adsorption equilibrium resulting from the saturation of the active adsorption sites, at 90 min for native cedar sawdust with an average adsorption rate of 71% and at 60 min for activated sawdust with an average adsorption rate of 97%.

The adsorbent’s mass has a positive effect on the adsorption rates of Cr (VI), which increase proportionally with the amount of sawdust. When the mass of native sawdust varies from 0.25 g to 2 g, the adsorption rates increase from 33% to 66% for native cedar. However, for 1 g of activated sawdust, the maximum adsorption rate is 93%, and then it stabilizes at this level. The increase in adsorption capacity with the mass of the adsorbent is due to the availability of a sufficient number of active sites responsible for the binding of Cr (VI). 

The temperature also has a positive effect on the adsorption rates of Cr (VI), which rise from 30% to 71% for native cedar sawdust and from 55% to 98% for activated cedar sawdust when the temperature changes from 25 to 50°C, respectively.

These results can be explained by the fact that the increase in the temperature of the reaction medium accelerates the mobility of the chromate ions (HCrO_4_^−^) in solution, thus promoting their fixation on the active sites of adsorption, so this is a question of an endothermic process. Similar results have been reported in previous studies that focus on the adsorption of Cr (VI) on biomass [46,47].

**Figure 6:**
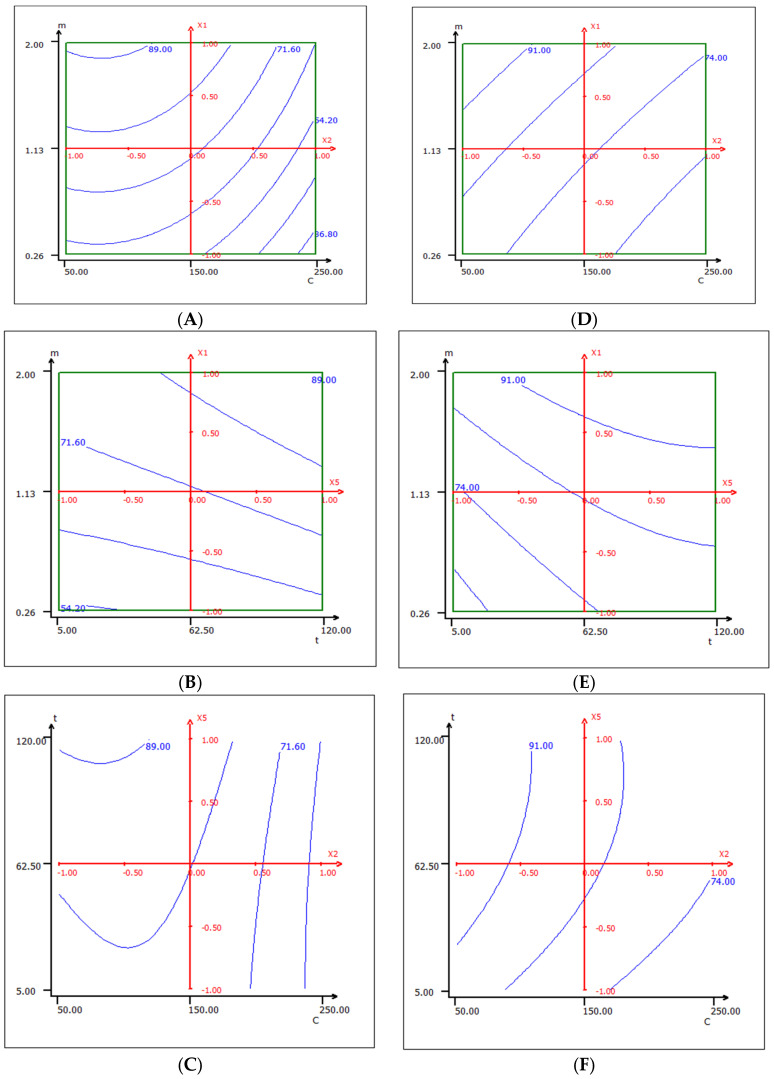
Variation of the adsorption rates of Cr (VI) in 2D in the plane (m, c): (**A**), (m, t): (**B**), and (t, c): (**C**) for native cedar and in the plane (m, c): (**D**), (m, t): (**E**), and (t, c): (**F**) for activated cedar (pH = 1, T = 50 °C g, and t = 67.5 min).

To achieve the maximum adsorption of Cr (VI) ions onto native and activated cedar sawdust, RSM modeling was used. The adsorption efficiency of native and modified cedar sawdust over different combinations of independent variables was visualized through 2D and 3D views of response surface plots (Figure 6A–E), which are represented as a function of two parameters at a time, while holding other factors at a fixed level. These plots were used to determine the individual and cumulative effect of the factors, and the mutual interaction between them and the dependent factor.

The 2D graphs illustrate the isoreponse curves that show the adsorption rate of Cr (VI) ions onto native cedar (Figure 6A–C) and activated cedar (Figure 6D–F), depending on the level of the parameters. Moving towards areas of low concentration and high values of contact time, it is found that the adsorption rate of Cr (VI) ions increases, while it decreases for the highest concentrations and the lowest contact times. 

**Figure 7:**
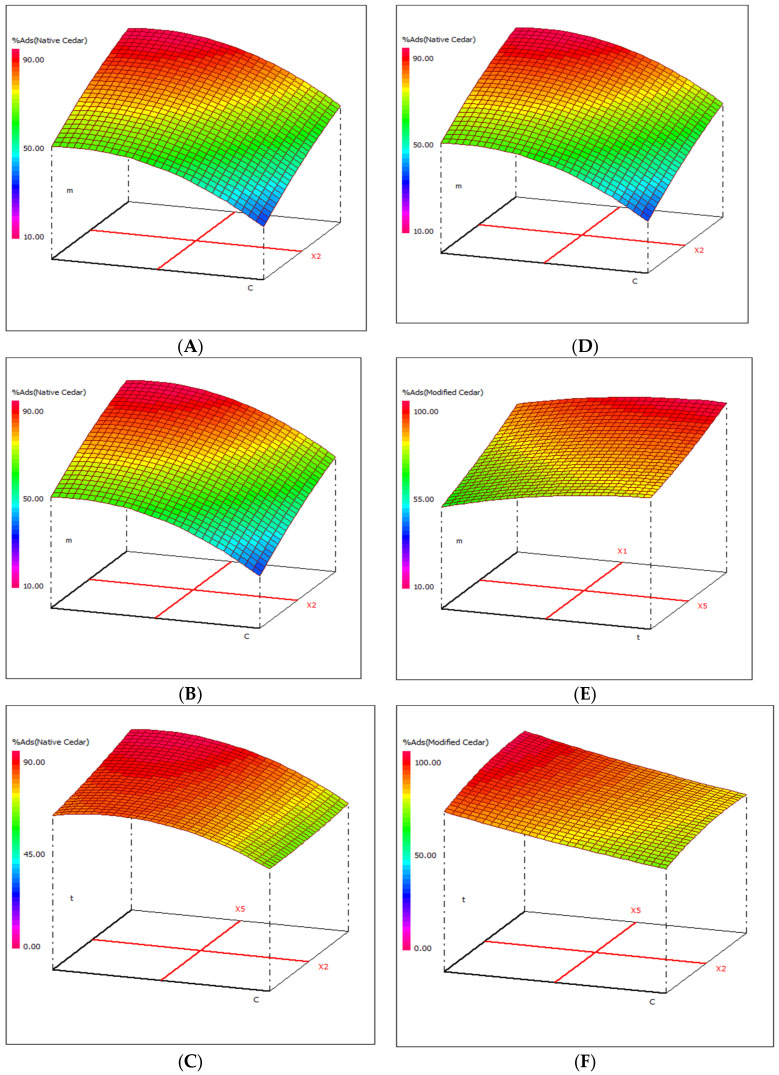
Variation of the adsorption rates of Cr (VI) in 3D in the plane (m, c): (**A**), (m, t): (**B**), and (t, c): (**C**) for native cedar and in the plane (m, c): (**D**), (m, t): (**E**), and (t, c): (**F**) for activated cedar (pH = 1, T = 50 °C g, and t = 67.5 min).

The 3D graphical presentations for the adsorption of Cr (VI) on native cedar (Figure 7A–C) and on activated cedar (Figure 7D–F) reveal response surfaces where the red color expresses the surface with a higher adsorption rate of Cr (VI) ions. It is found that the optimal conditions for reaching maximum adsorption rates are to carry out adsorption, in the case of native Cedar, at high levels (+1) of pH and contact time, and at central levels (0) of adsorbent mass and metal concentration, and in the case of activated cedar, at a high level (+1) of pH and at central levels (0) of adsorbent mass, contact time, and metal concentration.

#### 2.2.4. Experimental Validity Test: Test Point

As the last step of the optimization process, we have applied the validity test point of the postulated model in order to obtain a result corresponding to the desired response (Cr (VI) adsorption rate). The optimal values of the various factors are collated in Table 4.

The obtained results show that there is no significant difference between the experimental responses and those predicted using the experimental design model. Therefore, the chosen model perfectly maps the Cr (VI) adsorption process onto the native and modified cedar sawdusts. 

#### 2.2.5. Kinetics of Cr (VI) Adsorption

In order to study the fixation kinetics of Cr (VI) on the surfaces of native and modified cedar sawdust, two models were used, namely the pseudo-first-order model ($Ln(q_{e}-q_{t})=Ln(q_{e})-k_{1}\cdot t$) and the pseudo-second-order model ($\frac{t}{q_{t}}=\frac{1}{k_{2}\cdot q_{e}^{2}}+\frac{t}{q_{e}}$) [48,49,50].

**Figure 8:**
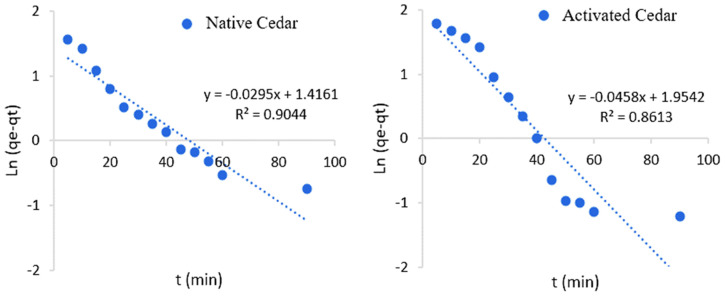
Curves illustrating the pseudo-first-order kinetic model for Cr (VI) adsorption on native and modified cedar sawdust.

**Figure 9:**
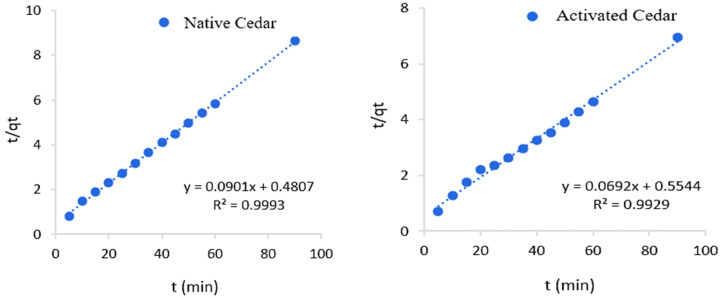
Curves illustrating the pseudo-second-order kinetic model for Cr (VI) adsorption on native and modified cedar sawdust

The obtained results are shown in Figure 8 and Figure 9, and the kinetic constants deduced from these graphs are collated in Table 5.

From the results of the linear regression analysis of the two models and the correlation coefficients corresponding to the experimental results, it can be seen that the Cr (VI) adsorption system is in agreement with the pseudo-second-order model, with a k_2_ of 0.01 g/mg.min in the case of modified cedar and 0.02 g/mg.min in the case of native cedar. This is explained by the high values of the coefficients of determination (R^2^ > 0.99) and the agreement between the maximum calculated adsorption capacities (*q_e_* cal) and those obtained experimentally (*q_e_* exp).

#### 2.2.6. Isotherms of Cr (VI) Adsorption

The study of the Cr (VI) adsorption isotherm on native and modified cedar sawdust was carried out under the optimal conditions described above, by plotting the curve of the quantity adsorbed in equilibrium (*q_e_*) as a function of the equilibrium concentration (*C_e_*).

The experimental adsorption curves were correlated with the Langmuir ($\frac{1}{q_{e}}=\frac{1}{q_{\max}}+\frac{1}{k_{l}C_{e}q_{\max}}$) and Freundlich ($Log(q_{e})=Log(k_{f})+{\frac{1}{n}}_{f}Log(c_{e})$) models [48,49,50].

**Figure 10:**
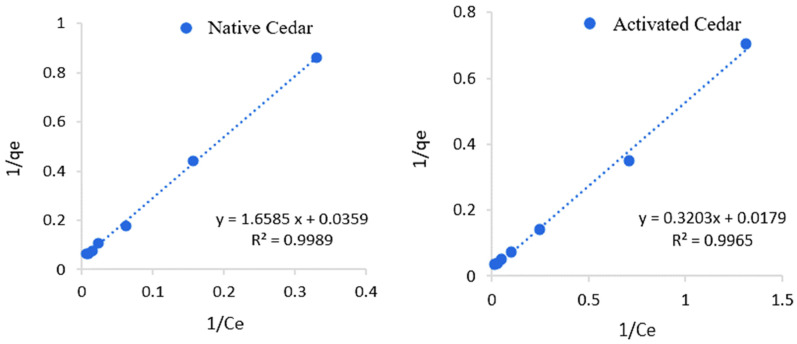
Representations of Langmuir isotherms describing the adsorption of Cr (VI) on the different sawdusts.

**Figure 11:**
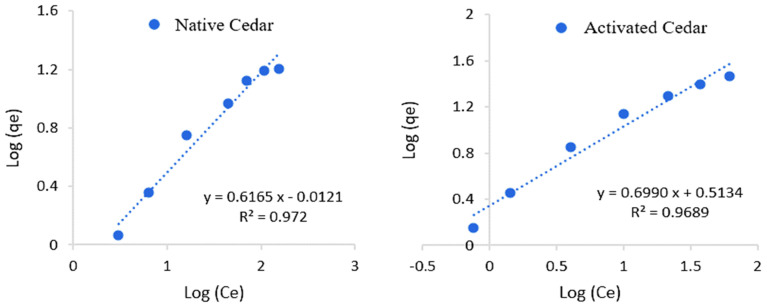
Representations of Freundlich isotherms describing the adsorption of Cr (VI) on the different sawdusts.

**Figure 12:**
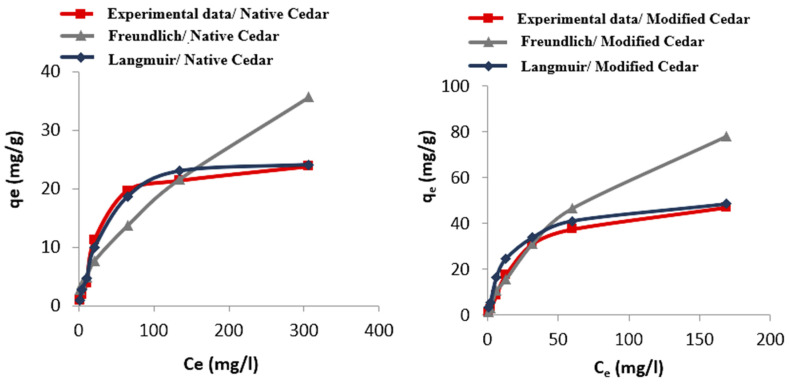
Adsorption isotherms of Cr (VI) on native and modified cedar using Langmuir and Freundlich models.

The linear shapes of the isotherms and the experimental adsorption isotherms are represented in Figure 10, Figure 11 and Figure 12, and the parameters calculated for these two models are grouped together in Table 6.

The Cr (VI) adsorption process on native and modified cedar sawdust follows the Langmuir model with high coefficients of determination (R² > 0.99). This model assumes that adsorption is monolayered and occurs at homogeneous and specific sites. The adsorption capacity of Cr (VI) (*q*_max_) on modified sawdust is greater than that obtained with native sawdust: 48.31 mg/g for activated cedar and 23.64 mg/g for native cedar. The Langmuir constant (*k_l_*) is 0.04 L/mg in the case of modified cedar and 0.02 L.mg in the case of native cedar, which means that there is more affinity with or strength of the modified cedar for Cr (VI) adsorption; in another words, a higher kl indicates a stronger binding between the solute and the adsorbent surface.

### 2.3. Possible Mechanisms of Cr (VI) Adsorption onto Native and Modified Sawdust

The Cr (VI) adsorption mechanism using native or modified sawdust involves different physical and chemical processes. Sawdust, as a lignocellulosic material, contains functional groups such as hydroxyl (-OH), carboxyl (-COOH), and phenolic groups on its surface, which can interact with Cr (VI) ions through adsorption processes. A detailed explanation of the possible mechanisms involved in Cr (VI) adsorption onto cedar is as follows:

1. Chemisorption: Cr (VI) ions can undergo chemisorption onto the surface of sawdust through covalent bonding. The oxygen-containing functional groups on sawdust, such as hydroxyl and carboxyl groups, can form strong bonds with Cr (VI) ions, leading to their immobilization on the surface.
2. Electrostatic interaction: Cr (VI) ions are anionic species in aqueous solutions. The positively charged functional groups on the sawdust surface, such as protonated amino groups or other positively charged sites, can electrostatically attract and adsorb the negatively charged Cr (VI) ions.
3. Ion exchange: Sawdust contains various cations, which can undergo ion exchange with Cr (VI) ions in the solution. Cr (VI) ions can replace these cations on the sawdust surface through ion exchange mechanisms, leading to the adsorption of Cr (VI) ions. The ion exchange capacity of sawdust is influenced by the pH of the solution. At lower pH values, more H+ ions are available for exchange, while at higher pH values, competition with other anions may reduce ion exchange efficiency.
4. Reduction: Sawdust may contain reducing agents or compounds that can facilitate the reduction of Cr (VI) to Cr (III). Cr (VI) reduction to Cr (III) can take place on the surface of sawdust, promoting the adsorption of Cr (III) ions, which are less toxic and less soluble than Cr (VI) ions.
5. Complexation: Functional groups on sawdust, such as phenolic groups, can form complexes with Cr (VI) ions. Complexation involves the formation of stable coordination compounds between the functional groups on the sawdust surface and Cr (VI) ions, leading to their adsorption.
6. Physical adsorption: Apart from chemical interactions, physical adsorption also plays a role. Van der Waals forces and other weak interactions can attract Cr (VI) ions onto the surface of sawdust, contributing to the overall adsorption process.

The effectiveness of Cr (VI) adsorption onto native and modified sawdust depends on several factors, including the characteristics of the adsorbent (particle size, surface functional groups), experimental conditions (pH, temperature), the initial concentration of Cr (VI) in the solution, and the contact time between the adsorbate and the adsorbent. Therefore, it is our role as researchers to conduct experiments and use mathematical models to optimize these parameters for efficient Cr (VI) removal using sawdust as an adsorbent. 

Based on the findings of this study, the suggested mechanisms are chemisorption and ion exchange as the isotherm modeling was found to follow the Langmuir model, which suggests that the adsorption is carried out on a single layer using chemical bonding, and this was also confirmed by the saturation plateau which happened after a certain contact time. The effect of pH clearly showed that at low pH values, the adsorption was at a maximum because of the presence of more available H+ ions for exchange.

## 3. Materials and Methods

### 3.1. Preparation and Modification of the Biosorbent

The Moroccan Atlas cedar sawdust used in this study was first sieved, and part of the fraction between 100 and 500 µm was used in the experiments as a native biosorbent. 

The other part of the fraction was used to prepare a biosorbent modified with 20% nitric acid and sodium hydroxide (1 N), following the protocol described in our previous study [51].

### 3.2. Characterization of the Biosorbent

The structure of native and modified Atlas cedar sawdusts were characterized in terms of the detection of functional groups using Fourier Transform Infrared Spectroscopy (Perkin Elmer brand FTIR spectrometer) over the spectral range of 400–4000 cm^−1^ using KBr wafers. The morphology of these adsorbents was imaged via Scanning Electron Microscopy (Philips XL30I SEM). The change in crystallinity was examined via X-ray diffraction analysis using a powder diffractometer (XPERT-PRO Philips XRD) within the range of 2*θ* = 5–40° with Cu-Kα radiation (k = 1.54 Å).

### 3.3. Adsorption Process Based on a Batch System

A 1000 mg/L stock solution of Cr (VI) was prepared by dissolving potassium dichromate (K_2_Cr_2_O_7_). Subsequently, synthetic wastewater samples were prepared by progressively diluting the stock solutions with distilled water to achieve the desired concentrations. To achieve the desired pH levels, small volumes of 0.1 M HNO_3_ and NaOH solutions were added to the prepared solutions.

The adsorption trials were carried out by adding a known quantity of the adsorbents to 100 mL of Cr (VI) in 150 mL closed glass bottles. Different experiments were performed to study the effect of adsorbent’s mass (m), concentration of Cr (VI) (C_0_), temperature (T), pH (pH), and contact time (t) on the Cr (VI) removal percentages. Each batch adsorption test generated via the RSM design based on the CCD program was carried out using fixed magnetic agitation and the samples were separated via centrifugation prior to any analysis. After a certain contact time, the residual concentration of Cr (VI) was analyzed via flame atomic absorption spectrometry (AAS) using a GBC 932 AA spectrophotometer. The adsorbed quantity of Cr (VI) (*q_e_*) and removal percentages of Cr (VI) (%Ads) were calculated using Equations (3) and (4) [51].

(3) $$q_{e}=\frac{c_{0}-c_{e}}{m}\times V$$

(4) $$\%Ads=\frac{c_{0}-c_{e}}{c_{0}}\times 100$$

where *q_e_* (mg/g) is the adsorbed quantity, C_0_ (mg/L) is the initial concentration of Cr (VI) and *C_e_* (mg/L) is the final concentration of Cr (VI), V (L) is the volume of solution, and *m* (g) is the quantity of native and modified Atlas cedar.

### 3.4. Experimental Design Approach and Optimization

To estimate the effects of several factors on the adsorption capacity, the selection of an adequate experimental design is the most crucial key allowing us to avoid the traditional “one-factor-at-a-time” method. To examine the interactions of two or more parameters, response surface methodology (RSM) has been shown to be a beneficial and practical tool which studies the responses of various parameters by varying them simultaneously with minimal number of trials. RSM is known as a statistical procedure that utilizes quantitative data from appropriate trials to find the optimum combinations of factors and to obtain an optimal response. Varieties of factorial designs are available to accomplish this task. In this study, we used central composite design (CCD), as it is the most popular and successful design in the RSM [24,30,52], to evaluate the interactive effect of the different parameters and to determinate the optimum conditions for maximum Cr (VI) removal using native and modified Atlas cedar. 

The effects of five independent variables, namely, adsorbent dose (X_1_), initial Cr (VI) concentration (X_2_), temperature (X_3_), pH (X_4_), and contact time (X_5_) on Cr (VI) adsorption were studied using the batch technique of 54 experiments in total (repetition included) (Equation (5)): 32 fractional points, 20 axial points, and 2 points at center (zero level), the replicates in the experiments allow us to evaluate the pure error.

N = (2^5-1^ + (2 × 5) + 1) × 2 = 54 (5)

The matrix is varied at 3 levels: the higher level (+1), the lower level (−1), and center point (0). The adsorption rate (%) was considered the dependent variable. In the optimization step, the response can be related to the independent factors via quadratic polynomial equation according to Equation (6) given below [30]:

(6) $$Y=b_{0}+\sum_{i}b_{i}X_{i}+\sum_{i}b_{ii}X_{i}^{2}+\sum_{i\neq j}b_{ij}X_{ij}$$

where, *Y* is the adsorption rate, *b*_0_ is the model constant, *b_i_* is the linear coefficient, *b_ii_* are the quadratic coefficients and *b_ij_* are interaction coefficients between two of the four factors’ coefficients, and *X_i_* are the independent variables [24]. 

Experimental data were evaluated with the NemrodW program, which was created in 1972 by Professor Roger Phan-Tan-Luu. It is an essential tool for constructing a wide range of optimal experiment matrices and for analyzing experiment results. During the model fitting process, various statistical analysis techniques were employed to assess factors such as experimental error, model appropriateness, and the statistical significance of the model’s components. All of these analyses were conducted within the same program, and one of the techniques used was ANOVA (Analysis of Variance). ANOVA was utilized to confirm the suitability of the quadratic model and to evaluate the significance of each variable based on its respective *p*-value [53,54].

The quality of the fit achieved by the quadratic model is conveyed by the determination coefficient, denoted as the R^2^ value. R^2^ values serve as a reliable metric for gauging how effectively the observed variations in response values can be elucidated by the experimental factors and their interactions [55,56]. The significance of R^2^ is derived from its ability to offer insights into the model’s goodness of fit and the fraction of the dependent variable’s variability that can be attributed to the independent variables. A high R^2^ value, approaching 1, signifies that a substantial portion of the variability in the dependent variable is explained by the independent variables. This suggests that the model effectively represents the data and excels in making predictions.

Each parameter’s field, shown in Table 1, was defined based on our previous work [51]. Every parameter was tested on three levels as shown in Table 7 (low level (−1), central level (0), and high level (+1)), including the interactions between these three levels. 

### 3.5. Adsorption Modeling 

The kinetic study was monitored using the pseudo-first-order (Equation (7)) and pseudo-second-order (Equation (8)) models:

(7) $$Ln(q_{e}-q_{t})=Ln(q_{e})-k_{1}t$$

(8) $$\frac{t}{q_{t}}=\frac{1}{k_{2}\times q_{e}^{2}}+\frac{1}{q_{e}}t$$

The isotherms were modeled according to the Langmuir (Equation (9)) and Freundlich (Equation (10)) models:

(9) $$\frac{1}{q_{e}}=\frac{1}{q_{\max}}+\frac{1}{klC_{e}q_{\max}}$$

(10) $$Log(q_{e})=Log(k_{f})+{\frac{1}{n}}_{f}Log(c_{e})$$

## 4. Conclusions

The adsorption study of Cr (VI) on native and HNO_3_/NaOH-activated cedar sawdust made it possible to evaluate the capacity and the efficiency of these sawdusts at retaining the Cr (VI) ions from a solution using atomic absorption spectroscopy. 

Several factors have been optimized, namely, adsorbent dosage (m), chrome (VI) concentration (Co), temperature (T), pH, and contact time (t) through the application of an experimental design methodology in order to predict the optimal conditions for the adsorption of Cr (VI) ions on these sawdusts. The finding of this optimization shows that the optimal experimental conditions to eliminate the maximum amount of Cr (VI) are a pH of 1, sawdust mass of 2 g for native cedar and 1.125 g for activated cedar, metal concentration of 150 mg/L for native cedar and 250 mg/L for its activated form, temperature of 50 °C, and a contact time of 67.5 min, which were enough to achieve Cr (VI) adsorption rates of around 85% on the native cedar and around 99% on its activated form. These results prove that the adsorption process of Cr (VI) on both native and activated cedar sawdust was effective. 

The modeling of the adsorption isotherms of Cr (VI) on both forms of sawdust follows the Langmuir model (R^2^ > 0.99). The maximum adsorbed quantities determined experimentally are close to those determined theoretically. The adsorption kinetics follow the pseudo-second-order model with determination coefficients R^2^ very close to unity.

This study has shed light on the effective use of sawdust as an adsorbent for heavy metals, demonstrating its potential in the depollution of industrial effluents loaded with Cr (VI). As we look towards the future, our next steps will focus on the regeneration of these sawdusts and similar absorbent materials. By refining the regeneration process, we aim to enhance the efficiency and longevity of these materials. Subsequently, we plan to harness these regenerated materials in the development of hybrid construction materials.

## Figures and Tables

**Table 1:** Design matrix in terms of real and coded values of response (Cr (VI) removal) and the experimental results of CCD.

Exp N°	M	C_0_	T	pH	t	%Ads (Native Cedar)	%Ads (Modified Cedar)
	g	mg/L	°C	--	min	%	%
1	0.25	50.00	25.00	1.00	120.00	45.00	62.00
2	0.25	50.00	25.00	1.00	120.00	30.00	48.00
3	2.00	50.00	25.00	1.00	5.00	58.00	61.00
4	2.00	50.00	25.00	1.00	5.00	35.00	58.00
5	0.25	250.00	25.00	1.00	5.00	40.00	41.00
6	0.25	250.00	25.00	1.00	5.00	20.00	29.00
7	2.00	250.00	25.00	1.00	120.00	42.00	42.00
8	2.00	250.00	25.00	1.00	120.00	28.00	33.00
9	0.25	50.00	50.00	1.00	5.00	56.00	67.00
10	0.25	50.00	50.00	1.00	5.00	48.00	52.00
11	2.00	50.00	50.00	1.00	120.00	98.00	100.00
12	2.00	50.00	50.00	1.00	120.00	75.00	89.00
13	0.25	250.00	50.00	1.00	120.00	36.00	62.00
14	0.25	250.00	50.00	1.00	120.00	23.00	51.00
15	2.00	250.00	50.00	1.00	5.00	72.00	78.00
16	2.00	250.00	50.00	1.00	5.00	44.00	52.00
17	0.25	50.00	25.00	6.00	5.00	13.00	30.00
18	0.25	50.00	25.00	6.00	5.00	4.00	8.00
19	2.00	50.00	25.00	6.00	120.00	23.00	30.00
20	2.00	50.00	25.00	6.00	120.00	6.00	12.00
21	0.25	250.00	25.00	6.00	120.00	9.00	28.00
22	0.25	250.00	25.00	6.00	120.00	2.00	6.00
23	2.00	250.00	25.00	6.00	5.00	17.00	29.00
24	2.00	250.00	25.00	6.00	5.00	9.00	7.00
25	0.25	50.00	50.00	6.00	120.00	29.00	30.00
26	0.25	50.00	50.00	6.00	120.00	11.00	19.00
27	2.00	50.00	50.00	6.00	5.00	30.00	43.00
28	2.00	50.00	50.00	6.00	5.00	16.00	23.00
29	0.25	250.00	50.00	6.00	5.00	10.00	17.00
30	0.25	250.00	50.00	6.00	5.00	6.00	13.00
31	2.00	250.00	50.00	6.00	120.00	29.00	35.00
32	2.00	250.00	50.00	6.00	120.00	20.00	25.00
33	0.25	150.00	37.50	3.50	62.50	50.00	81.00
34	0.25	150.00	37.50	3.50	62.50	39.00	70.00
35	2.00	150.00	37.50	3.50	62.50	75.00	99.00
36	2.00	150.00	37.50	3.50	62.50	52.00	78.00
37	1.125	50.00	37.50	3.50	62.50	60.00	98.00
38	1.125	50.00	37.50	3.50	62.50	45.00	82.00
39	1.125	250.00	37.50	3.50	62.50	45.00	85.00
40	1.125	250.00	37.50	3.50	62.50	37.00	62.00
41	1.125	150.00	25.00	3.50	62.50	31.00	36.00
42	1.125	150.00	25.00	3.50	62.50	29.00	32.00
43	1.125	150.00	50.00	3.50	62.50	89.00	93.00
44	1.125	150.00	50.00	3.50	62.50	65.00	80.00
45	1.125	150.00	37.50	1.00	62.50	75.00	100.00
46	1.125	150.00	37.50	1.00	62.50	52.00	74.00
47	1.125	150.00	37.50	6.00	62.50	27.00	40.00
47	1.125	150.00	37.50	6.00	62.50	13.00	29.00
49	1.125	150.00	37.50	3.50	5.00	55.00	78.00
50	1.125	150.00	37.50	3.50	5.00	44.00	58.00
51	1.125	150.00	37.50	3.50	120.00	74.00	96.00
51	1.125	150.00	37.50	3.50	120.00	56.00	77.00
53	1.125	150.00	37.50	3.50	62.50	86.00	93.00
54	1.125	150.00	37.50	3.50	62.50	64.00	72.00

**Table 2:** Effect of the factors influencing the adsorption of Cr (VI) onto native and modified cedar and their coefficients (***: highly significant coefficient, **: very significant coefficient, * significant coefficient).

Coef.	Effect		t. Experimental		Signification (*p*-Value)	
	Native Cedar	Modified Cedar	Native Cedar	Modified Cedar	Native Cedar	Modified Cedar
b_0_	58.30	80.776	17.17	23.77	<0.01 ***	<0.01 ***
b_1_	7.16	5.000	3.33	2.32	0.216 **	2.67 *
b_2_	−5.36	−6.028	−2.49	−2.80	1.80 *	0.856 **
b_3_	8.78	9.361	4.07	4.34	0.0272 ***	0.0126 ***
b_4_	−16.75	−18.750	−7.78	−8.70	<0.01 ***	<0.01 ***
b_5_	1.64	2.806	0.76	1.30	0.0452 ***	0.02 ***
b_1-1_	−2.21	1.439	−0.38	0.25	70.8	80.7
b_2-2_	−9.46	1.189	−1.62	0.20	11.5	84.0
b_3-3_	−2.71	−20.311	−0.46	−3.47	64.6	0.146 **
b_4-4_	−14.46	−19.811	−2.47	−3.39	1.87 *	0.184 **
b_5-5_	1.04	−3.311	0.18	−0.57	86.0	0.575 *
b_1-2_	0.31	1.438	0.14	−0.63	1.809 *	0.05 ***
b_1-3_	3.44	3.563	1.50	1.56	14.2	12.9
b_2-3_	−2.37	0.125	−1.04	0.05	30.6	95.7
b_1-4_	−2.75	−1.500	−1.20	−0.66	23.7	51.6
b_2-4_	3.44	3.563	1.50	1.56	14.2	12.9
b_3-4_	−2.69	−3.813	−1.18	−1.67	24.8	10.5
b_1-5_	1.62	1.063	0.71	−0.46	0.482 **	0.0645 ***
b_2-5_	−2.69	−1.000	−1.18	−0.44	0.248 **	0.665 **
b_3-5_	1.56	2.125	0.68	0.93	49.9	35.9
b_4-5_	0.62	−1.263	0.27	−0.66	78.6	64.5

**Table 3:** Analysis of variance for the fitted model for the %Ads of Cr (VI) on native and modified cedar sawdust.

Source of Variation	Sum of Squares		Degrees of Freedom	Mean Square		Rapport		Signif. *p*-Value	
	Native Cedar	Modified Cedar		Native Cedar	Modified Cedar	Native Cedar	Modified Cedar	Native Cedar	Modified Cedar
Regression	2.5 × 10^4^	3.6 × 10^4^	20	1.3 × 10^3^	1.8 × 10^3^	7.51	10.92	<0.01	<0.01
Residual	5.5 × 10^3^	5.5 × 10^3^	33	1.6 × 10^2^	1.6 × 10^2^				
Validity	1.9 × 10^3^	1.7 × 10^3^	6	3.2 × 10^2^	2.8 × 10^2^	2.42	2.11	5.3	8.5
Error	3.6 × 10^3^	3.7 × 10^3^	27	1.3 × 10^2^	1.4 × 10^2^				
Total	3.1 × 10^4^	4.2 × 10^4^	53						
R²	0.82 (Native Cedar), 0.88 (Modified Cedar)								
R²Adj	0.74 (Native Cedar), 0.79 (Modified Cedar)								

**Table 4:** Predicted and experimental values for the test points: Cr (VI) adsorption on native and activated cedar sawdust.

	Parameters	Value	Code	Predicted Response (%)	Experimental Response (%)
Native Cedar	m (g)	2	+1	83	84.16
	C (ppm)	150	0		
	T (°C)	50	+1		
	pH	1	−1		
	t (min)	62.5	0		
Modified Cedar	m (g)	1.125	0	100	99.04
	C (ppm)	250	+1		
	T (°C)	50	+1		
	pH	1	−1		
	t (min)	62.5	0		

**Table 5:** Kinetic parameters of the pseudo-first-order and pseudo-second-order models.

Sawdust	Pseudo-First-Order Model				Pseudo-Second-Order Model			
	R^2^	*k*_1_ (min^−1^)	*q_e_* cal (mg/g)	*q_e_* exp (mg/g)	R^2^	*k*_2_ (g/mg.min)	*q_e_* cal (mg/g)	*q_e_* exp (mg/g)
Native Cedar	0.90	0.03	4.12	10.89	0.99	0.02	11.10	10.89
Modified Cedar	0.86	0.05	7.06	13.24	0.99	0.01	14.45	13.24

**Table 6:** Langmuir and Freundlich constants relating to the adsorption process of Cr (VI) on native and modified cedar sawdust.

Sawdust	Langmuir Model			Freundlich Model		
	R^2^	*q*_max_ (mg/g)	*k_l_* (L/mg)	R^2^	*k_f_*	n
Native Cedar	0.99	23.64	0.02	0.97	0.65	1.47
Modified Cedar	0.99	48.31	0.04	0.96	2.22	1.46

**Table 7:** Experimental field of selected parameters.

Designation	Notation	Low Level (−1)	Central Level (0)	High Level (+1)
X_1_	Mass: m (g)	0.25	1.125	2
X_2_	Concentration: C (mg/L)	50	150	250
X_3_	Temperature: T (°C)	25	37.5	50
X_4_	pH	1	3.5	6
X_5_	Contact time: t (min)	15	67.5	120

## Data Availability

All the data in the article are available from the corresponding author upon reasonable request.

## Sample Availability

Samples of the compounds are not available from the authors.

## References

[1] Bagbi Y., Sarswat A., Mohan D., Pandey A., Solanki P.R. (2016). Lead (Pb^2+^) Adsorption by Monodispersed Magnetite Nanoparticles: Surface Analysis and Effects of Solution Chemistry. J. Environ. Chem. Eng..

[2] Fu F., Wang Q. (2011). Removal of Heavy Metal Ions from Wastewaters: A Review. J. Environ. Manag. J..

[3] Yousefzadeh H., Salarian A.A., Sid Kalal H. (2018). Study of Pb (II) Adsorption from Aqueous Solutions by TiO_2_ Functionalized with Hydroxide Ethyl Aniline (PHEA/n-TiO_2_). J. Mol. Liq..

[4] Al-Shahrani S.S. (2014). Treatment of Wastewater Contaminated with Cobalt Using Saudi Activated Bentonite. Alex. Eng. J..

[5] Gupta V.K., Ali I. (2004). Removal of Lead and Chromium from Wastewater Using Bagasse Fly Ash—A Sugar Industry Waste. J. Colloid. Interfaces Sci..

[6] Thakur R., Sharma G.D., Dwivedi B.S., Khatik S.K. (2007). Chromium: As a Pollutant. J. Ind. Pollut. Control.

[7] Du J., Shang X., Shi J., Guan Y. (2022). Removal of Chromium from Industrial Wastewater by Magnetic Flocculation Treatment: Experimental Studies and PSO-BP Modelling. JWPE.

[8] Karegar S., Bhargavi M., Divekqr S.V. (2015). Treatment of Wastewater from Chrome Plating Industry by Ion Exchange Method. IJRET.

[9] Mella B., Glanert A.C.C., Gutterres M. Removal of Chromium from Tanning Wastewater by Chemical Precipitation and Electrocoagulation. Proceedings of the XXXII. Congress of IULTCS.

[10] Wen J., Sun Y., Ning P., Xu G., Sun S., Sun Z., Cao H. (2022). Deep Understanding of Sustainable Vanadium Recovery from Chrome Vanadium Slag: Promotive Action of Competitive Chromium Species for Vanadium Solvent Extraction. J. Hazard. Mater..

[11] Sowmya C., Purnima D. (2022). Chrome Removal from Bulk Drug Industry Effluent Using Fly Ash Waste Generated in Industrial Process. Mater. Today Proc..

[12] Ramakul P., Yanachawakul Y., Leepipatpiboon N., Sunsandee N. (2012). Biosorption of Palladium(II) and Platinum(IV) from Aqueous Solution Using Tannin from Indian Almond (*Terminalia catappa* L.) Leaf Biomass: Kinetic and Equilibrium Studies. J. Chem. Eng..

[13] Mutiara T., Setyaningsih L., Chafidz A., Panandita B.S., Raharjo R. (2019). Alkali Modified Jackfruit Wood Sawdust as Bio Adsorbent for Removal of Pb(II) Ions from Wastewaters. IOP Conf. Ser. Mater. Sci. Eng..

[14] Gao Z., Liu Q., Wang H., Xia N., Zhu S. (2019). Adsorption Mechanism of Cu(II) and Pb(II) from Aqueous Solutions Using Citric Acid Modified Beet Pulp Fiber (CDSBP) and Fe-Modified CDSBP. Desalin. Water Treat..

[15] Yi Z.J., Yao J., Chen H.L., Wang F., Liu X., Xu J.S. (2016). Equilibrium and Kinetic Studies on Adsorption of Pb(II) by Activated Palm Kernel Husk Carbon. Desalin. Water Treat. Water Treat..

[16] Mitra T., Bar N., Das S.K. (2019). Rice Husk: Green Adsorbent for Pb(II) and Cr(VI) Removal from Aqueous Solution—Column Study and GA–NN Modeling. SN Appl. Sci..

[17] Larous S., Meniai A.H., Bencheikh Lehocine M. (2005). Experimental Study of the Removal of Copper from Aqueous Solutions by Adsorption Using Sawdust. Desalination.

[18] Boudy P. (1955). Economie Forestière Nord Africaine: Monographie et Traitement Des Essences Résineuses. Tome II Fascicule 2.

[19] M’Hirit O., Blerot P. (1999). Le Grand Livre de La Forêt Marocaine.

[20] Uehara A., Tommis B., Belhassen E., Satrani B., Ghanmi M., Baldovini N. (2017). Odor-Active Constituents of Cedrus Atlantica Wood Essential Oil. Phytochem..

[21] Bezerra M.A., Santelli R.E., Oliveira E.P., Villar L.S., Escaleira L.A. (2008). Response Surface Methodology (RSM) as a Tool for Optimization in Analytical Chemistry. Talanta.

[22] Bhunia P., Ghangrekar M.M. (2008). Statistical Modeling and Optimization of Biomass Granulation and COD Removal in UASB Reactors Treating Low Strength Wastewaters. Bioresour. Technol..

[23] Ren J., Lin W.T., Shen Y.J., Wang J.F., Luo X.C., Xie M.Q. (2008). Optimization of Fermentation Media for Nitrite Oxidizing Bacteria Using Sequential Statistical Design. Bioresour. Technol..

[24] Öztürk D., Şahan T. (2015). Design and Optimization of Cu(II) Adsorption Conditions from Aqueous Solutions by Low-Cost Adsorbent Pumice with Response Surface Methodology. Pol. J. Env. Stud..

[25] Su S.N., Nie H.L., Zhu L.M., Chen T.X. (2009). Optimization of Adsorption Conditions of Papain on Dye Affinity Membrane Using Response Surface Methodology. Bioresour. Technol..

[26] Guo W.Q., Ren N.Q., Wang X.J., Xiang W.S., Ding J., You Y., Liu B.F. (2009). Optimization of Culture Conditions for Hydrogen Production by Ethanoligenens Harbinense B49 Using Response Surface Methodology. Bioresour. Technol..

[27] Bajpai S., Gupta S.K., Dey A., Jha M.K., Bajpai V., Joshi S., Gupta A. (2012). Application of Central Composite Design Approach for Removal of Chromium (VI) from Aqueous Solution Using Weakly Anionic Resin: Modeling, Optimization, and Study of Interactive Variables. J. Hazard. Mater..

[28] Gunaraj V., Murugan N. (1999). Application of Response Surface Methodology for Predicting Weld Bead Quality in Submerged Arc Welding of Pipes. J. Mater. Process. Technol..

[29] Adinarayana K., Ellaiah P. (2002). Response Surface Optimization of the Critical Medium Components for the Production of Alkaline Protease by a Newly Isolated *Bacillus* sp. J. Pharm. Pharm. Sci..

[30] Şahan T., Öztürk D. (2014). Investigation of Pb(II) Adsorption onto Pumice Samples: Application of Optimization Method Based on Fractional Factorial Design and Response Surface Methodology. Clean. Technol. Env. Policy.

[31] El Hajam M., Idrissi Kandri N., Harrach A., El khomsi A., Zerouale A. (2019). Physicochemical Characterization of Softwood Waste “Cedar” and Hardwood Waste “Mahogany”: Comparative Study. Mater. Today Proc..

[32] El Hajam M., Kandri N.I., Zerouale A., Wang X., Gustafsson J., Wang L., Mäkilä E., Hupa L., Xu C. (2022). Lignocellulosic Nanocrystals from Sawmill Waste as Biotemplates for Free-Surfactant Synthesis of Photocatalytically Active Porous Silica. ACS Appl. Mater. Interfaces.

[33] Kalavathy M.H., Regupathi I., Pillai M.G., Miranda L.R. (2009). Modelling, Analysis and Optimization of Adsorption Parameters for H3PO4 Activated Rubber Wood Sawdust Using Response Surface Methodology (RSM). Colloids Surf. B.

[34] Singh K.P., Gupta S., Singh A.K., Sinha S. (2011). Optimizing Adsorption of Crystal Violet Dye from Water by Magnetic Nanocomposite Using Response Surface Modeling Approach. J. Hazard. Mater..

[35] Khezeli T., Daneshfar A. (2015). Monodisperse Silica Nanoparticles Coated with Gold Nanoparticles as a Sorbent for the Extraction of Phenol and Dihydroxybenzenes from Water Samples Based on Dispersive Micro-Solid-Phase Extraction: Response Surface Methodology. J. Sep. Sci..

[36] Ravikumar K., Krishnan S., Ramalingam S., Balu K. (2007). Optimization of Process Variables by the Application of Response Surface Methodology for Dye Removal Using a Novel Adsorbent. Dye. Pigm..

[37] Jing X., Cao Y., Zhang X., Wang D., Wu X., Xu H. (2011). Biosorption of Cr(VI) from Simulated Wastewater Using a Cationic Surfactant Modified Spent Mushroom. Desalination.

[38] Sereshti H., Entezari Heravi Y., Samadi S. (2012). Optimized Ultrasound-Assisted Emulsification Microextraction for Simultaneous Trace Multielement Determination of Heavy Metals in Real Water Samples by ICP-OES. Talanta.

[39] Ravikumar K., Pakshirajan K., Swaminathan T., Balu K. (2005). Optimization of Batch Process Parameters Using Response Surface Methodology for Dye Removal by a Novel Adsorbent. J. Chem.Eng..

[40] Şahan T., Ceylan H., Aktaş N. (2016). Optimization of Biosorption of Zn(II) Ions from Aqueous Solutions with Low-Cost Biomass Trametes Versicolor and the Evaluation of Kinetic and Thermodynamic Parameters. Desalin. Water Treat..

[41] Baral S.S., Das S.N., Rath P. (2006). Hexavalent Chromium Removal from Aqueous Solution by Adsorption on Treated Sawdust. Biochem. Eng. J..

[42] Ahalya N., Kanamadi R.D., Ramachandra T. (2008). V Biosorption of Chromium (VI) by Tamarindus Indica Pod Shells. JESRI.

[43] Ramos R.L., Martinez A.J., Guerrero Coronado R.M. (1994). Adsorption of Chromium (VI) from Aqueous Solutions on Activated Carbon. Water Sci. Tech..

[44] Ucun H., Bayhan Y.K., Kaya Y., Cakici A., Faruk Algur O. (2002). Biosorption of Chromium (VI) from Aqueous Solution by Cone Biomass of Pinus Sylvestris. Bioresour. Technol..

[45] Park D., Yun Y.S., Park J.M. (2005). Use of Dead Fungal Biomass for the Detoxification of Hexavalent Chromium: Screening and Kinetics. Process Biochem..

[46] Arica M.Y., Bayramoǧlu G. (2005). Cr (VI) Biosorption from Aqueous Solutions Using Free and Immobilized Biomass of Lentinus Sajor-Caju: Preparation and Kinetic Characterization. Colloids Surf. A.

[47] Shukla A., Zhang Y.-H., Dubey P., Margrave J.L., Shukla S.S. (2002). The Role of Sawdust in the Removal of Unwanted Materials from Water. J. Hazard. Mater..

[48] El Hajam M., Idrissi Kandri N., Harrach A., El khomsi A., Zerouale A. (2019). Adsorption of Methylene Blue on Industrial Softwood Waste “Cedar” and Hardwood Waste “Mahogany”: Comparative Study. Mater. Today Proc..

[49] El Hajam M., Idrissi Kandri N., Zerouale A. (2019). Batch Adsorption of Brilliant Green Dye on Raw Beech Sawdust: Equilibrium Isotherms and Kinetic Studies. Moroc. J. Chem..

[50] El Hajam M., Kandri N.I., Harrach A., Zerouale A. (2019). Adsorptive Removal of Brilliant Green Dye from Aqueous Solutions Using Cedar and Mahogany Sawdusts. Sci. Study Res..

[51] El Hajam M., Kandri N.I., Plavan G.I., Harrath A.H., Mansour L., Boufahja F., Zerouale A. (2020). Pb2+ Ions Adsorption onto Raw and Chemically Activated Dibetou Sawdust: Application of Experimental Designs. J. King Saud. Univ. Sci..

[52] Anupam K., Dutta S., Bhattacharjee C., Datta S. (2011). Adsorptive Removal of Chromium (VI) from Aqueous Solution over Powdered Activated Carbon: Optimisation through Response Surface Methodology. J. Chem.Eng..

[53] Kütahyali C., Sert Ş., Çetinkaya B., Yalçintaş E., Acar M.B. (2012). Biosorption of Ce(III) onto Modified Pinus Brutia Leaf Powder Using Central Composite Design. Wood Sci. Technol..

[54] Asadollahzadeh M., Tavakoli H., Torab-Mostaedi M., Hosseini G., Hemmati A. (2014). Response Surface Methodology Based on Central Composite Design as a Chemometric Tool for Optimization of Dispersive-Solidification Liquid-Liquid Microextraction for Speciation of Inorganic Arsenic in Environmental Water Samples. Talanta.

[55] Yetilmezsoy K., Demirel S., Vanderbei R.J. (2009). Response Surface Modeling of Pb(II) Removal from Aqueous Solution by Pistacia Vera L.: Box-Behnken Experimental Design. J. Chem. Eng..

[56] Khajeh M. (2011). Optimization of Process Variables for Essential Oil Components from Satureja Hortensis by Supercritical Fluid Extraction Using Box-Behnken Experimental Design. J. Supercrit. Fluids.

